# The Dimensions of Pet-Owner Loyalty and the Relationship with Communication, Trust, Commitment and Perceived Value

**DOI:** 10.3390/vetsci5040095

**Published:** 2018-11-06

**Authors:** Bryan R. Brown

**Affiliations:** College of Business, Wilmington University, 320 N. DuPont Hwy, New Castle, DE 19720, USA; bbrown019@my.wilmu.edu; Tel.: +1-757-708-1989

**Keywords:** customer satisfaction, customer loyalty, attitudinal loyalty, behavioral loyalty, relationship between satisfaction and loyalty, communication, trust, commitment, perceived value, value co-creation, veterinarian, veterinary medicine, pet-owner

## Abstract

Loyalty is one of the greatest intangible assets that any organization can possess and improving client loyalty is a primary marketing goal that can have a significant financial impact on any business. This quantitative study examined the mediating role of communication on the relationship between satisfaction and loyalty (attitudinal and behavioral) in veterinary clinics, along with the moderating roles of trust, commitment, perceived value, and relational characteristics. Responses collected from 351 pet-owners through social media were analyzed using descriptive and inferential statistics. The results show that attitudinal loyalty (AL) has a strong positive relationship with communication at multiple points in a veterinary clinic, whereas the relationship with behavioral loyalty (BL) was not as clear. Additional findings suggest that AL, which is influenced by trust in the veterinarian, communication from staff members and commitment, has a strong positive relationship with behavioral intentions, increases the number of products and services that a pet-owner consumes at his or her primary veterinary clinic, and attenuates the role of cost in receiving veterinary care. These findings can help veterinary clinic owners and managers in developing and implementing relationship strategies that improve pet-owner loyalty.

## 1. Introduction

The following article is a synopsis of the author’s dissertation [1]. Veterinary practice owners face a myriad of challenges in operating a successful business enterprise. Pet-owner adherence with recommended treatments fell from 29% to 22% between 2013 and 2016, while annual visits by pet-owners dropped from 4.4 to 3.2 [2,3]. The number of current pet-owners who report not taking pets to a veterinary clinic has risen steadily [4], corresponding with a decline in the number of new pet-owners visiting veterinary practices [5]. Up to 15% of pet-owners have defected from traditional veterinary clinics to an increasing number of alternative veterinary care delivery outlets including non-profit, mobile and retail-store providers [6]. These trends place extra importance on a veterinary clinic owner’s ability to improve pet-owner loyalty towards the practice. This exploratory study investigated whether a relationship exists between pet-owners’ satisfaction with the communication that is received at a veterinary clinic and loyalty expressed to the veterinary clinic.

Pet-owner loyalty is not guaranteed. Pet-owners, like any other consumer will change service providers in the pursuit of higher service quality and better value [7]. Often, a consumer will measure service quality on how the experience with a business made him or her feel, rather than what service was actually provided [8], and these positive feelings lead to overall satisfaction with a service provider [9]. Cumulative satisfactory experiences define pet-owner expectations about future interactions with a veterinarian, develop trust and influence loyalty [10]. However, understanding the nature of loyalty is not necessarily straightforward. The Veterinary Hospital Managers Association (VHMA) asked a group of veterinary practice managers to describe how pet-owners demonstrate loyalty [11]. Selected answers describe a client who: “regularly returns”, “provides referrals, and positive reviews”, “pays for whatever a pet needs” and “follows recommendations”. This diverse set of characteristics of loyalty provides a foundation of clinic expectations for client loyalty, but a further examination of the dimensions of loyalty is warranted.

Loyalty is one of the best intangible assets that an organization can have [12], thus defining loyalty accurately is of great importance. Multiple definitions of loyalty exist within the literature. The examples listed above by veterinary practice managers refer to actions that may be taken by pet-owners, yet a commitment to future actions must also be considered as a component of loyalty [13]. Thus the concept of relationship commitment implies continued loyalty by a pet-owner in the future [14] and must be part of any working definition. Loyalty to an organization begins with simple awareness of the organization. Commitment to further develop the relationship with the firm implies an affective component to loyalty in addition to a behavioral component [10]. To fully understand loyalty, each component must be examined.

Pet-owners may become aware of and choose a veterinary practice for any number of reasons; location, referral and price are the most common reasons cited [4]. Pet-owners may continue to patronize a veterinary practice for a long time simply out of familiarity [15]. After some period of time, these pet-owners might be considered to be a loyal customer of the veterinary practice. If the loyalty exhibited by the pet-owner centers entirely around awareness of the veterinary clinic, convenience and habit, it can be best described as cognitive loyalty. And as long as the pet-owner perceives little differentiation between the veterinary practice at which he or she currently frequents, and another practice, the pet-owner is not likely to move towards higher forms of loyalty. Oliver [13] describes this as “phantom loyalty” (p. 37) because consumers exhibiting this type of loyalty are more likely to switch service providers for any number of reasons including price and convenient location. As long as a minimum amount of customer satisfaction is maintained by the veterinary practice, the pet-owner will be less likely to find another practice, but satisfaction alone is not adequate to keep these pet-owners loyal. Up to 85% of patients in one study in the optometric industry who defected to other service providers stated that they were satisfied with the service which they had received [16], and there is no reason to believe that the veterinary health industry would be any different [13]. It is necessary for a veterinary practice to differentiate itself in some manner to move pet-owners beyond cognitive loyalty.

Attitudinal loyalty (AL) has affective and possibly emotive components that are lacking in cognitive loyalty [13]. As such, it should be considered more of a psychological construct than a behavioral construct [12]. Pet-owners who express AL are more inclined to utilize one veterinary clinic exclusively [17], are more likely to forgive service failures [18], may be willing to pay premium prices [19], and are willing to proactively recommend a veterinary practice to a friend [20]. Those who express AL are likely to commit psychologically to revisit the same veterinary practice [21]. Each of these benefits of AL is important to the profitability of a veterinary practice. AL should be cultivated and encouraged within a veterinary practice, but the veterinary practice owner must understand that attitude does not always mean action on the part of the pet-owner [17]. 

Behavioral loyalty (BL) is a function of purchase activity over time [22]. BL can exist with or without AL but combining the two can lead to firm profitability in a number of ways. Future revenue streams can be secured through improved loyalty [23], expenses can decrease due to reduced costs of new client acquisition [24], and job satisfaction within a veterinary clinic can increase with improved loyalty [25]. BL is, in essence, pet-owners adhering with the healthcare recommendations of a veterinarian at a single veterinary practice over the course of time. 

Achieving loyalty is a primary marketing goal that can have a significant financial impact on any business [23]. It has been estimated that acquiring a new customer is between five and 25 times more expensive than retaining an existing one, and that increasing customer retention rates by 5% can increase profits by 25% to 95% [26]. One study found that up to 40% of the variance in profitability between divisions of the same financial institution were attributable to customer loyalty [20]. While no studies could be found that specifically investigated the relationship between loyalty and profitability of a veterinary clinic, it does appear that this relationship occurs across many industries [27]. However, this relationship is not linear and at some point a business will see diminishing returns on investments in loyalty [28]. But overall, increasing the length of a business relationship can be one component of loyalty that will significantly improve the financial performance of a business. Many researchers have investigated whether there is a direct satisfaction between loyalty and profitability chain with mixed results [29,30,31,32] partially because, while loyal pet-owners are most likely satisfied, it cannot be said that all satisfied pet-owners are loyal. 

Pet-owner satisfaction with veterinarians is consistently very high, and in some cases it is above 90% [33], yet only 81.2% of pet-owners that self-describe as having a regular veterinarian [4]. Indeed, past research has shown that satisfaction does not always have a direct effect on loyalty [30], but is instead moderated by factors such as trust, commitment, and relational value [34] of which an important mediator is effective communication [32,35]. In a previous study, when pet-owners were asked ‘Why would you switch your veterinarian?’ the most common reasons given, other than price, were confusion, uncertainty, and misunderstanding about treatment recommendations [33]. Each of these secondary reasons could be eliminated with effective communication, and the primary reason of price could be attenuated with effective communication about the value of veterinary care.

Communication has been studied in human medicine for decades [36], and satisfaction with communication has been shown to improve patient retention and adherence, as well as being considered an overall positive health outcome [37]. However, the study of communication in veterinary medicine is a relatively new discipline [36]. Failure to effectively communicate can lead to client attrition [38] and improving communication has been shown to significantly increase pet-owner adherence with recommended treatments [39,40,41,42]. This increase in adherence benefits the health of the pet and the financial performance of the practice, yet many pet-owners do not understand the value of many of the recommendations made by veterinarians. In one survey, 36% of pet-owners stated that they only take a pet to the veterinarians to get vaccinations, 32% stated that they only take the pet to the veterinarian if the pet gets sick, and 24% stated that routine examinations are unnecessary [33]. This reflects a general lack of understanding of the value of preventive care for a pet that must be overcome in order to improve adherence with recommended treatments. 

The current cross-sectional study is exploratory and investigated whether pet-owners’ satisfaction with the communication that they received at a veterinary clinic had a relationship with both AL and BL to the veterinary clinic. The importance of this study is to better understand what pet-owners want, how they think, how their preferences can be turned into perceived value by the pet-owner, and ultimately to improve loyalty towards veterinary practices. The findings from this research serve as a baseline of pet-owner loyalty.

## 2. Materials and Methods

### 2.1. Participants

The target population for the survey was broadly defined as pet-owners who participate in one of two social media sites on which the survey was administered. A convenience sample of participants was gathered using a snowball methodology [43], whereby the author provided a link to the survey instrument to 20 individuals with whom the author maintains a professional or academic relationship. Those individuals then provided the link to his or her respective contact list on Facebook and LinkedIn along with a request to complete the survey. 

### 2.2. Instrumentation

The questionnaire consisted of four main sections in addition to demographics. The first section asked pet-owners to cite the frequency with which he or she observed customer service and communication behaviors from veterinary clinic staff members. This section was subdivided to include the front desk staff, veterinary technicians, veterinarians, and check-out staff. A four-point Likert scale was used with choices of rarely, sometimes, often, and always. Questions were selected from a client satisfaction survey from AAHA [44] for the non-veterinarian staff members and from a validated instrument designed to measure trust in the veterinarian [45]. The second section measured pet-owners perception of the cost of veterinary services using a four point-Likert scale from strongly disagree to strongly agree. The third section measured pet-owners’ attitudinal loyalty, commitment and overall satisfaction with communication using a 4-point Likert scale from strongly disagree to strongly agree. A validated instrument [10] measured AL. The fourth section asked pet-owners to identify whether he or she consumes a veterinary product or service along with the location of acquisition. A set of 8 commonly utilized products and services were selected from a list of commonly utilized products and services [4], and through consultation with practicing veterinarians. 

After the survey instrument was developed, it was sent to 12 industry experts for evaluation of content validity. Three of the experts provided feedback and each agreed that the instrument was valid for measuring communication, trust, value and loyalty. Minor changes were made to the wording of several of the questions based on the feedback that was provided. 

The final survey instrument was created using an online survey tool and was tested for reliability by the researcher. It was designed to collect primarily quantitative data. A four-point scale was chosen to eliminate the midpoint choice and to minimize central tendency bias after determining that a four-point scale would not adversely affect the reliability of the findings [46]. Opportunities were provided for participants to provide qualitative data and to elaborate on several questions by including “other” or “why” options on selected questions. Internal reliability was confirmed by conducting a Cronbach’s alpha test. The results of this test showed that each construct was above the desired minimum of 0.70 [47].

### 2.3. Analysis

Descriptive and inferential statistics were used to analyze the data using Statistical Package for the Social Science (SPSS) version 25 (IBM, Armonk, NY, USA). Answers to the qualitative questions were compiled and categorized by the author. A Spearman’s rho correlation was run to assess the relationship between pet-owner satisfaction with communication and the individual communication points, as well as with the higher-order constructs of trust, loyalty, commitment, and perception of value. To better understand the relationships, linear regressions were run on the higher order constructs. A logistic binomial regression was run for behavioral loyalty since it is a dichotomous construct. One-way analysis of variance (ANOVA) and independent sample *t*-tests were conducted to test the differences between various groups of pet-owners. All statistical analyses were overseen by a faculty statistician at Wilmington University.

### 2.4. Ethical Approval

This research received ethical approval from the Wilmington University Human Subjects Review Committee (HSRC). Participants were presented with a privacy disclosure and were required to agree to the terms in order to access the survey instrument. No data were collected to identify individual participants.

## 3. Results

A total of 435 individuals began the survey, and 351 (80.7%) of these individuals completed the survey. Partially completed surveys were omitted from the results. Because the survey was anonymous, it was not possible to determine the reasons for survey abandonment. The gender makeup of the population shows that 284 (80.1%) were female and 54 (15.4%) were male and 13 (4.5%) declined to self-identify. Completed surveys were received from 39 states and one Canadian province. The mean age of the population was 45.6. Respondents have patronized their current veterinarian for an average of 7.7 years. The mean number of pets for dog owners (*n* = 295, 84.0%) was 1.78, for cat owners (*n* = 143, 40.7%) was 1.47; some respondents reported owning both dogs and cats (*n* = 102, 29.1%). Pet-owners consider their pets to be members of the family (85.4%). This figure is higher than the 63.2% previously reported [4]. This could be an indicator that the current study population has a more favorable opinion of their pets, or it could reflect a general trend over time in the pet-owner population [48]. Pet-owners most commonly visit a veterinarian annually (*n* = 115, 33.24%). Some pet-owners reported that he or she takes pets to the veterinarian only when the pet is sick (*n* = 42, 12.14%) or when the pet needs a vaccination (*n* = 42, 12.14%). This is consistent with 10% of pet-owners completely agreeing that they only take a pet to the veterinarian when the pet is sick or needs a vaccination [33].

### 3.1. Pet-Owner Defection Rate

A total of 140 respondents (40.6%) reported having stopped patronizing a veterinary clinic at some point and provided reasons for leaving. Cost of care was the most commonly reported reason for leaving (*n* = 30, 21.4%), followed by a perception of poor care (*n* = 25, 17.9%). It is doubtful that lay pet-owners can accurately assess the quality of care that is provided by a veterinarian [45]; therefore, this reason must be listed as merely a perception. Customer service was directly cited by 20 pet-owners. However, categorizing reasons for defection indicates that 51.4% of pet-owner defections are directly attributable to some customer service related issue (see Table 1). In terms of customer service, perception is reality and, therefore, this perception by pet-owners must be acknowledged and addressed [8,49,50]. 

### 3.2. Satisfaction with Communication

Satisfaction is a highly personalized and subjective measure of past performance by a firm. Indeed, client satisfaction is what each client says it is [50], and most pet-owners agree or strongly agree that they are satisfied with communication from veterinarians (*n* = 318, 90.6%) and from veterinary clinic staff (*n* = 304, 86.6%). Satisfaction was compared to the communication point constructs to verify whether the constructs were measuring satisfaction with communication at each separate communication point. A Spearman’s rho correlation was run to assess the relationship between pet-owner satisfaction with communication and the individual communication points (see Table 2). 

### 3.3. Trust in the Veterinarian

Customer satisfaction alone is not enough to explain loyalty; without a foundation of trust and commitment, satisfaction can hardly improve loyalty for a firm in a significant way [30]. Trust is an outcome of sustained satisfaction [14] and is considered a key antecedent of relationship commitment [19]. As such, trust provides pet-owners with confidence of future reliability of services and satisfaction. Trust also mediates perceptions of value [51] and can influence the depth and breadth of service usage, which are important indicators of BL [52]. 

A linear regression was run to better understand the predictors of pet-owner trust in the veterinarian. Trust has been identified as an antecedent of both commitment and loyalty [53]. Therefore, the model for trust should exclude commitment and loyalty. The results of the regression indicated that three predictors explained 52% of the variance in trust in the veterinarian (R^2^ = 0.519, F_(3, 350)_ = 124.835, *p* < 0.001). It was found that the customer service and communication skills of veterinary technicians significantly predicted trust in the veterinarian (B = 0.345, *p* < 0.001), as did satisfaction with communication from the veterinarian (B = 0.286, *p* < 0.001), and customer service and communication skills from the checkout staff (B = 0.093, *p* = 0.013). Additionally, there is a strong positive relationship (R = 0.720) between trust and the model predictors. The linear regression model is statistically significant (*p* < 0.001).

### 3.4. Satisfaction to Loyalty

Numerous studies in a variety of industries have concluded that a relationship exists between satisfaction and loyalty [16,27,32]. This relationship is typically influenced by one or more moderators including trust [35,38], value [12,54], and commitment [10,55]. A summary of the relationship between each of these constructs for the present study is presented in Table 2. The findings showed that a strong positive relationship exists between AL and the many of the higher order constructs. These findings are consistent with Ball et al. [38], and Gounaris et al. [55]. Both of the aforementioned studies went on to conclude that trust and perceived value moderated their findings. Therefore, these variables were considered in the present study as well. Each had moderate positive relationships with AL. 

#### 3.4.1. Attitudinal Loyalty (AL)

To better understand the nature of the relationships above, a linear regression was conducted to investigate the predictors of AL. Results of the regression indicated that three predictors explained 81% of the variance in AL (R^2^ = 0.812, F_(3, 349)_ = 498.105, *p* < 0.001). It was found that trust in the veterinarian predicted AL (B = 0.164, *p* < 0.001), as did satisfaction with communication from the staff (B = 0.172, *p* < 0.001), and commitment to return to the practice (B = 0.640, *p* < 0.001). There is a strong positive relationship (R = 0.901) between AL and the model predictors. The linear regression model is statistically significant (*p* < 0.001). There was no evidence of multicollinearity as evidenced by a variance inflation factor (VIF) of less than five for each variable. The significance of the potential impact of commitment on this model reflects its strong relationship with AL that was found in Table 2, it also reflected the findings of previous research [10,55].

Commitment to conduct future business is a key moderator of loyalty and can be thought of as a cognitive intention towards future BL. As such, it is important to understand the factors that can influence commitment. A linear regression was performed with two models. The first model investigated commitment in the absence of AL. In this model, the regression indicated that five predictors explained 69% of the variance in pet-owners’ commitment to return to the practice (R^2^ = 0.685, F_(5, 349)_ = 149.295, *p* < 0.001). It was found that trust in the veterinarian predicted pet-owner commitment (B = 0.158, *p = 0*.009), as did satisfaction with communication from the veterinarian (B = 0.490, *p* < 0.001), satisfaction with communication from the staff (B = 0.215, *p* < 0.001), the perception of cost and value of services (B = 0.196, *p* < 0.001), and satisfaction with the checkout process (B = −0.071, *p* = 0.015). There is a strong positive relationship (R = 0.827) between commitment and the model predictors. The linear regression model is statistically significant (*p* < 0.001). 

Model two introduced AL to the commitment linear regression model. It has been shown that commitment is not only a predictor of AL, but is also moderated by AL [30]. A pet-owner who has developed AL with a clinic would be influenced more by model two. In this model, the regression indicated that two predictors explained 82% of the variance in pet-owners’ commitment to return to the practice (R^2^ = 0.817, F_(2, 349)_, *p* < 0.001). It was found that satisfaction with communication from the veterinarian significantly predicted pet-owner commitment (B = 0.296, *p* < 0.001), as did AL (B = 0.699, *p* < 0.001). Additionally, there is a strong positive relationship (R = 0.904) between commitment and the model predictors. The linear regression model is statistically significant (*p* < 0.001).

#### 3.4.2. Behavioral Loyalty (BL)

BL was determined to exist with a pet-owner who patronized the same veterinary clinic for a minimum of five years and consumed at least five of eight identified products or services contained in the construct Behavioral Loyalty at the pet-owner’s primary veterinary clinic. A total of 57% of pet-owners have frequented his or her current veterinary clinic for a minimum of five years. Pet-owners consume an average of 5.95 (1.79) products or services from his or her primary veterinarian, and 81.8% of pet-owners consume at least five services at his or her primary veterinary clinic. In total, 47.6% of all pet-owners were deemed to be behaviorally loyal based on the criteria presented above.

BL has a weak relationship with many of the higher order constructs (see Table 2). To understand the components of BL, a binomial logistic regression was performed. The logistic regression model was statistically significant, χ^2^_(13)_ = 47.086, *p* < 0.001, and explained 20% (Nagelkerke R^2^) of the variance in behavioral loyalty and correctly classified 66.8% of cases. Sensitivity was 69.0%, specificity was 64.6%, positive predictive value was 66.2% and negative predictive value was 67.4%. The positive predictive value was calculated as the percentage of correctly predicted number of pet-owners exhibiting behavioral loyalty compared to the total number of pet-owners who were predicted as having behavioral loyalty. The negative predictive value was calculated as the percentage of correctly predicted number of pet-owners who were not behaviorally loyal compared to the total number of pet-owners who were not behaviorally loyal.

Of the 13 variables considered for the model, only age (pet-owner), satisfaction with the customer service and communication of the technician, and satisfaction with communication from the staff were statistically significant. One observation of note in this model is that a pet-owner who is satisfied with the communication of the staff is nearly twice as likely to be behaviorally loyal than a pet-owner who is not satisfied with the communication of the staff, Exp(B) = 1.975. A Hosmer and Lemeshow goodness-of-fit test was performed on the model. It demonstrated that the model chosen was not a poor fit but failed to determine whether the model was a good fit. 

### 3.5. Perceptions of Value

The cost of veterinary services was cited as the leading reason for a pet-owner leaving a veterinary clinic, it is therefore important to understand this construct further. A one-way ANOVA was conducted to determine whether there was a difference in the perception of cost and value of services between any of the demographic groups, or constructs. A number of variables had statistically significant differences between groups, including commitment to return, satisfaction with veterinarian communication, satisfied with staff communication, AL, and perceived importance of wellness exams. Prior to conducting the ANOVA, the mean score for pet-owners’ perception of cost and value was binned into three categories. A score of 1.0 to 1.9 corresponds to a response of “strongly disagree”. A score of 2.0 to 2.9 represents a response of “disagree”. A score of 3.0 to 4.0 represents a response of “agree” or “strongly agree”. Results of the ANOVA show statistically significant differences across each of the five variables in terms of satisfaction with cost and value of veterinary services (*p* < 0.001). A Tukey post hoc analysis revealed that mean increase in satisfaction with cost and value of services was statistically significant with each of the group differences (*p* < 0.001) with the exception of “up to 2.0” and “2.0 to 2.9” in the group “how important are routine checkups” (*p* = 0.167). 

To understand the predictors of pet-owners perception of cost and value, a linear regression was conducted. The results of the regression indicated that four predictors explained 52% of the variance (R^2^ = 0.521, F_(4, 338)_ = 90.653, *p* < 0.001). It was found that satisfaction with customer service and communication skills of the checkout staff significantly predicted pet-owners’ perception of cost and value (B = 0.235, *p* < 0.001), as did AL (B = 0.236, *p* < 0.001), satisfaction with communication from the veterinarian (B = 0.200, *p* < 0.001), and pet-owners’ perception of the importance of wellness exams (B = 0.128, *p* < 0.001). There is a strong positive relationship (R = 0.721) between cost and value, and the model predictors. The linear regression model is statistically significant (*p* < 0.001).

### 3.6. Age Differences

Differences in mean responses of the variables cost and value, trust in the veterinarian, AL, commitment to return, satisfaction with veterinarian communication, and satisfaction with staff communication were observed based on the age of the respondent. Ages were binned into four groups: up to 30, 31 to 45, 46 to 60, and 61 and older. Observationally, the mean for each variable increases through the first three age brackets before falling at the fourth (see Table 3). This finding warranted further investigation.

A one-way ANOVA was conducted to test the differences in the age brackets for the selected variables. The results show statistically significant differences between age groups for each of the variables. A Tukey post hoc test was conducted to identify which groups within the age brackets had statistically significant differences. The results of the Tukey post hoc analysis revealed that the youngest cohort of pet-owners is less satisfied with the communication that they receive from veterinarians and staff members alike. It also showed that they, along with the oldest cohort, are less likely to be committed to conducting further business with the veterinary clinic and have an overall lower AL score. This finding could have long-term consequences since the youngest cohort may be pet-owners for a long time to come.

### 3.7. Behavioral Effects of Attitudinal Loyalty

A veterinary practice’s ability to maintain a long-term, sustainable competitive advantage is tied to its ability to retain and grow its customer base. This can require nurturing the relationship beyond mere repurchase behavior [9], and this nurturing is reflected in AL. Yet maintaining a profitable veterinary practice requires purchase behaviors from pet-owners, or at least purchase behavior intentions [16]. This raises the question of what behavioral effects are associated with AL, if any? The present study was able to identify several behaviors and behavioral intentions associated with AL, although a noted limitation of the present study is that these behaviors are self-reported recall behaviors rather than actual documented measurements of behavior.

Behavioral intentions are motivational drivers that have impacts on future business outcomes [17]. Behavior intentions were measured in the present study in two ways. First, respondents were asked about his or her intention, or commitment to continue to use his or her primary veterinary clinic in the future. Second, respondents were asked whether he or she would refer other pet-owners to his or her primary veterinary clinic. A strong positive relationship exists between both measures of behavior intention and AL, as well as satisfaction with communication from the veterinarian and staff (see Table 2). A moderate positive relationship was found to exist between both measures of behavior intention and each communication point that was measured for the study.

One measure of BL in the present study is the number of products or services that were consumed by the pet-owner at his or her primary veterinary clinic. There is a weak statistically significant positive relationship between the number of products or services consumed at the pet-owners’ primary veterinary clinic with AL, r_s_ = 0.363, *p* < 0.001, and commitment, r_s_ = 0.309, *p* < 0.001. This offers an initial indication that attitude does have some relationship with behaviors. A series of independent sample t-tests were performed to determine whether there was a statistically significant difference in attitudinal loyalty for each product or service that was consumed.

Independent samples t-tests demonstrated that the differences in mean AL was statistically significantly higher for each of the eight services for pet-owners who received that product or service at their primary veterinary clinic than for those who did not. The differences were as follows: wellness exam, M = 0.52, 95% CI (0.21, 0.83), t_(349)_ = 3.339, *p* = 0.001; diagnostic laboratory services, M = 0.43, 95% CI (0.20, 0.65), t_(349)_ = 3.769, *p* < 0.001; vaccinations, M = 0.42, 95% CI (0.22, 0.63), t_(349)_ = 4.045, *p* < 0.001; prescription medications, M = 0.30, 95% CI (0.11, 0.48), t_(349)_ = 3.067, *p* = 0.002; other surgeries, M = 0.46, 95% CI (0.32, 0.61), t_(349)_ = 6.182, *p* < 0.001; spay or neuter surgery, M = 0.29, 95% CI (0.14, 0.43), t_(349)_ = 3.836, *p* < 0.001; preventive medications, M = 0.35, 95% CI (0.21, 0.49), t_(349)_ = 4.802, *p* < 0.001; dental procedure, M = 0.29, 95% CI (0.14, 0.43), t_(349)_ = 3.836, *p* < 0.001. While the t-tests confirmed statistically significant differences in AL scores between groups of pet-owners, it could not demonstrate a cause and effect relationship.

A linear regression was conducted to determine which variables, if any could predict the number of services that a pet-owner consumes at his or her primary veterinary clinic. Commitment was excluded from the model since its inclusion caused the variance inflation factor (VIF) to rise above 5.0 for AL, indicating multicollinearity. The results of the regression indicated that three predictors explained 20% of the variance (R^2^ = 0.201, F_(3, 271)_ = 22.448, *p* < 0.001). It was found that AL significantly predicted the number of products and services that a pet-owner consumes at his or her primary veterinary clinic (B = 0.938, *p* < 0.001), as did the pet-owners’ perception of the importance of wellness exams (B = 0.399, *p* < 0.001), and pet-owner income (B = 0.165, *p* < 0.001). There is a moderate positive relationship (R = 0.448) between the number of products and services consumed and the model contributors. The linear regression model is statistically significant (*p* < 0.001). 

## 4. Discussion

The present quantitative study investigated whether a relationship exists between pet-owners’ satisfaction with the communication that is received at a veterinary clinic and loyalty expressed to the veterinary clinic. The findings demonstrated that most pet-owners are satisfied with the communication that he or she receives at a veterinary clinic, and a positive relationship was established between this satisfaction and AL towards the veterinary clinic. Pet-owners in the present study also have a high level of AL, which means that pet-owners prefer using his or her current veterinary clinic in a way that is affective in nature. This bond is much more difficult for a competitor to sever than a bond that is purely transactional [9]. AL was demonstrated to influence many of the higher order constructs including trust in the veterinarian, perception of value in veterinary care, commitment to return, and the number of products or services consumed at the primary veterinary clinic. This finding highlights the positive aspect of developing pet-owners’ affective relationship with veterinary clinics. 

Several factors emerged as important moderators of AL and behavioral intentions of pet-owners. Those included the pet-owners’ perception of cost and value of services, whether the pet-owner sees the same veterinarian, the pet-owners’ perception of the importance of routine care, the age of the pet-owner, and whether the pet-owner consults a veterinarian or the internet first with questions about pet healthcare. Creating and maintaining AL, commitment, and even trust in the veterinarian was shown to involve all members of the veterinary clinic staff. This is confirmed by the inclusion of satisfaction with staff communication as a statistically significant contributor in each of these higher order constructs’ regression models. This finding emphasizes the need to ensure that each member of a veterinary clinic staff is providing a positive communication experience with pet-owners.

The relationship between satisfaction with communication and BL was not conclusive. While the relationship between BL and many of the higher order constructs was statistically significant, its relationship was weak at best. As noted earlier, it is possible that the definition of BL used in this research is flawed; it is also possible that the expectation of a strong relationship was unwarranted. A third option that must be considered is that the definition is correct, but sufficient satisfactory communication is not taking place within veterinary practices to affect BL. This possibility must be considered because BL did not appear as a statistically significant contributor to any of the higher order construct regression models, unlike AL which appeared in many. The understanding of BL will require further research.

The cost and perceived value of veterinary services are not in alignment. Most respondents (91.7%) agreed that the veterinary care that their pet received was good value for the money. However, nearly a third of respondents (30.1%) agreed or strongly agreed that veterinarians offer additional services just to make money. This finding suggests that the totality of care that a pet requires has not been fully explained in a way in which the pet-owner understands, or values. Pet-owners cannot be expected to understand all of the complexities of a physical exam [56], thus carefully and thoroughly explaining to a pet-owner what is involved in a thorough examination, followed by clear healthcare recommendations should improve perceived value. One study demonstrated that pet-owners are seven times more likely to follow a clear healthcare recommendation than a vague healthcare recommendation [57]. Offering a clear healthcare recommendation following a thoroughly explained physical examination might improve a pet-owner’s perception of the value and quality of care that his or her pet received, and could also improve BL. This would be beneficial for the veterinary clinic since poor quality of care was a highly cited as a reason for pet-owners’ defection. Framing this conversation around the health and wellbeing of pets would present medical information to pet-owners in the way in which they prefer to receive it [58].

Customer perceived value is independent of timing and can be considered a pre- or post-purchase construct [59]. Veterinary practices routinely set prices that are competitive in the marketplace for services that are commonly compared by prospective pet-owners in an effort to entice new pet-owners to become new clients with the practice [60]. This tactic could be effective in gaining new clients from local competition, but does not take into account products or services that are not commonly compared [61]. Pricing strategies could be developed that consider what matters most to the pet-owner. 

Customer service problems persist despite high overall pet-owner satisfaction with communication. This is highlighted by the high number of pet-owners who cited various deficiencies in customer service as reasons for defecting from a veterinary practice (see Table 1). A direct relationship between satisfaction with customer service and loyalty to a business is speculative at best [18]. But poor customer service is sure to decrease loyalty [50]. Every member of a veterinary clinic can affect perceptions about customer service. Additionally, it was noted earlier that every member of the clinic can affect loyalty, trust and commitment. Therefore, efforts to improve customer service should involve every member of a veterinary clinic.

Improvements in pet-owner loyalty can increase the frequency at which pet-owners visit a veterinary practice and improve adherence with recommended treatments [23]. The present research has demonstrated that satisfaction with communication has a significant positive relationship with attitudinal loyalty, which translates into higher perceived value in veterinary care, improved likelihood of positive behavioral intentions, and improvements in adherence with recommended treatments that are consumed at the primary veterinary clinic. 

There are several limitations noted within the study. The present study did not observe the hypothesized relationship between satisfaction with communication and behavioral loyalty. It is possible that no such relationship exists, but it is also possible that the current definition of behavioral loyalty must be refined, thus altering the findings. One factor that may have biased the findings on behavioral loyalty is that adherence data was self-reported by pet-owners. A more reliable source of adherence data could be extracted from a veterinary clinic’s database. Examining data directly from this database could address another limitation, which is a lack of veterinary clinic profitability. While an estimate can be made for the value of AL using the self-reported data, this estimate will remain speculative until further research can investigate this further. One other limitation is that pet-owners were solicited to participate in the current research through social media, and these individuals might not be reflective of the population as a whole.

Females made up 80.1% of the survey population. It may be difficult to translate the findings of this research to the population as a whole. However, this demographic profile is not unusual in studies in veterinary medicine. Other surveys found female participation at 84% [62], and 85% [63]. The percentage of women who self-report as being the primary care-giver to pets within households is 80.7% [4], indicating that the present findings may translate to pet-owner households.

## 5. Conclusions

The present exploratory study was able to confirm that a positive relationship does exist between satisfaction with communication and AL. This relationship also exists between trust in the veterinarian and attitudinal loyalty, as well as between the perceptions of cost and value of veterinary services, and attitudinal loyalty. The importance of AL has been demonstrated to be a predictor of a pet-owner’s perception of the cost and value of veterinary services along with the number of products or services that the pet-owner consumes at his or her primary veterinary clinic. AL was also shown to have a strong positive relationship with the behavioral intentions of commitment and willingness to refer. One of the strongest indicators of the value of AL comes in the examination of commitment.

It was noted that, in the absence of AL, pet-owner commitment to return to his or her present veterinary clinic is influenced by trust in the veterinarian, satisfaction with communication from the veterinarian and staff, and satisfaction with the cost and value of services. Once AL is introduced, pet-owner commitment to return is only influenced by AL and satisfaction with veterinarian communication. Eliminating the predictors of trust, satisfaction with staff communication, and perception of cost and value implies that pet-owners who exhibit AL might indeed be inclined to forgive service failures [18] as well as have a more favorable view of the cost and value of services [19]. Thus, developing and improving AL within pet-owners should have demonstrable benefits to veterinary clinics.

The relationship between satisfaction with communication and BL was not as clear. It was discovered that pet-owners who are satisfied with communication from staff members were nearly twice as likely to be BL, and that the likelihood of a pet-owner being behaviorally loyal improved when pet-owners see the same veterinarian and consult with the veterinarian first for healthcare questions. However, the full impact of BL on a veterinary clinic as measured for this study remains unclear.

Additional factors emerged as important moderators of attitudinal loyalty and behavioral intentions of pet-owners. Those included the pet-owners’ perception of cost and value of services, whether the pet-owner sees the same veterinarian, the pet-owners’ perception of the importance of routine care, the age of the pet-owner, and whether the pet-owner consults a veterinarian or the internet first with questions about pet healthcare.

Several areas for future research surfaced when evaluating this study. A better understanding of behavioral loyalty is required to fully understand the relationship between satisfaction with communication and behavioral loyalty. Gaining a better understanding could also help to determine the relationship between attitudinal loyalty and behavioral loyalty. 

The scope of the current research did not allow for the measurement of veterinary clinic profitability. It would be of great importance to understand the relationship between pet-owner loyalty, and practice profitability. Such a study would require the participation of one or more veterinary clinics to allow access to sensitive financial data at both a practice and pet-owner level. Using an information technology company that specializes in veterinary practices would allow for a more accurate measurement of the financial data and could serve to protect the anonymity of pet-owners.

Pet-owners generally agree that the veterinary care that is provided is good value for money, yet the cost of veterinary care is the most commonly cited reason for leaving a veterinary practice. This disconnect should be explored further to expand upon the present research and the qualitative research conducted by Coe et al. [58]. 

A commonly cited reason for pet-owners leaving a veterinary practice are the customer service skills of veterinarians and their staff members. Understanding what clients expect should be a priority for every veterinary practice and warrants further investigation. So too does the nature of the age differences in attitudinal loyalty that was uncovered in this research. It is necessary for veterinary clinic owners to fully understand the needs of the youngest cohort of pet-owners since they could own pets for a very long time.

The availability of loyalty and referral programs had no statistically significant relationship with any of the higher-order constructs in the present study. However, these types of programs have been successful in other industries [17,24]. These programs should be studied in a more focused way in the veterinary healthcare industry.

## Figures and Tables

**Table 1:** Pet owner reasons for not returning to veterinary clinic.

Reason	*n* = 140	%
Cost of Care	30	21.4
Perceived Poor Care	25	17.9
Veterinarian Personality	22	15.7
Customer Service	20	14.3
Moved	19	13.6
Staff Personality	10	7.1
Unkind Atmosphere	7	5.0
Undefined	7	5.0
Facilities (hours, dirty)	6	4.3
Lack of Empathy	6	4.3
Poor Communication	3	2.1
Veterinarian Retired	2	1.4
Death of Pet	2	1.4
Unresponsive	1	0.7
Death of Veterinarian	1	0.7
Facility Closed	1	0.7

**Table 2:** Spearman correlation: satisfaction with communication.

Variable	1	2	3	4	5	6	7	8	9	10	11	12	13
1 Satisfied Vet													
2 Satisfied Staff	0.826												
3 Front Desk	0.495	0.590											
4 Technicians	0.421	0.462	0.551										
5 Checkout Staff	0.497	0.607	0.789	0.626									
6 Checkout Process	0.406	0.401	0.442	0.378	0.457								
7 Attitudinal Loyalty	0.765	0.731	0.504	0.450	0.468	0.328							
8 Behavioral Loyalty	0.232	0.241	0.124	0.006	0.114	0.010	0.244						
9 Cost/Value	0.630	0.602	0.530	0.454	0.533	0.397	0.608	0.202					
10 Trust	0.564	0.491	0.452	0.535	0.481	0.340	0.562	0.206	0.502				
11 Services ^1^	0.285	0.271	0.201	0.090	0.125	0.069	0.363	0.400	0.301	0.158			
12 Willing to Refer ^2^	0.784	0.733	0.494	0.425	0.453	0.318	0.901	0.229	0.598	0.539	0.313		
13 Commitment	0.774	0.730	0.488	0.433	0.451	0.298	0.883	0.229	0.594	0.528	0.309	0.926	
14 Important Checkup	0.227	0.203	0.219	0.207	0.158	0.125	0.263	0.144	0.333	0.245	0.304	0.234	0.260

^1^ The number of products and services consumed by a pet-owner at his or her primary veterinary clinic. ^2^ The pet-owner’s willingness to refer other pet-owners to his or her primary veterinary clinic.

**Table 3:** Mean differences by age.

	≤30		31–45		46–60		61+	
	Mean	SD	Mean	SD	Mean	SD	Mean	SD
Cost and Value	2.65	0.56	2.93	0.55	3.07	0.61	2.88	0.58
Trust	3.54	0.54	3.65	0.51	3.78	0.41	3.75	0.46
Attitudinal Loyalty	3.00	0.64	3.29	0.72	3.52	0.61	3.19	0.64
Commitment	3.14	0.85	3.42	0.71	3.60	0.65	3.27	0.71
Satisfied Veterinarian	3.04	0.74	3.36	0.68	3.54	0.71	3.24	0.69
Satisfied Staff	3.00	0.77	3.27	0.75	3.44	0.72	3.21	0.65
